# DIEANet: an attention model for histopathological image grading of lung adenocarcinoma based on dimensional information embedding

**DOI:** 10.1038/s41598-024-56355-0

**Published:** 2024-03-14

**Authors:** Zexin Wang, Jing Gao, Min Li, Enguang Zuo, Chen Chen, Cheng Chen, Fei Liang, Xiaoyi Lv, Yuhua Ma

**Affiliations:** 1 College of Software, Xinjiang University, Urumqi, 830046 China; 2 Xinjiang Key Laboratory of Clinical Genetic Testing and Biomedical Information, Karamay, 834099 China; 3 Xinjiang Clinical Research Center for Precision Medicine of Digestive System Tumor, Karamay, 834099 China; 4 Department of Pathology, Karamay Central Hospital, Karamay, 834099 China; 5 College of Information Science and Engineering, Xinjiang University, Urumqi, 830046 China; 6 Key Laboratory of Signal Detection and Processing, Xinjiang University, Urumqi, 830046 China; 7 Xinjiang Cloud Computing Application Laboratory, Karamay, 834099 China

**Keywords:** Auxiliary diagnosis, Lung adenocarcinoma, Grading, Local information, Dimensional information, Lung cancer, Engineering, Computational biophysics

## Abstract

Efficient and rapid auxiliary diagnosis of different grades of lung adenocarcinoma is conducive to helping doctors accelerate individualized diagnosis and treatment processes, thus improving patient prognosis. Currently, there is often a problem of large intra-class differences and small inter-class differences between pathological images of lung adenocarcinoma tissues under different grades. If attention mechanisms such as Coordinate Attention (CA) are directly used for lung adenocarcinoma grading tasks, it is prone to excessive compression of feature information and overlooking the issue of information dependency within the same dimension. Therefore, we propose a Dimension Information Embedding Attention Network (DIEANet) for the task of lung adenocarcinoma grading. Specifically, we combine different pooling methods to automatically select local regions of key growth patterns such as lung adenocarcinoma cells, enhancing the model's focus on local information. Additionally, we employ an interactive fusion approach to concentrate feature information within the same dimension and across dimensions, thereby improving model performance. Extensive experiments have shown that under the condition of maintaining equal computational expenses, the accuracy of DIEANet with ResNet34 as the backbone reaches 88.19%, with an AUC of 96.61%, MCC of 81.71%, and Kappa of 81.16%. Compared to seven other attention mechanisms, it achieves state-of-the-art objective metrics. Additionally, it aligns more closely with the visual attention of pathology experts under subjective visual assessment.

## Introduction

The grading of invasive pulmonary adenocarcinoma (hereinafter referred to as lung adenocarcinoma) can guide physicians in developing treatment plans and patient prognosis^1–4^. The specific criteria of the grading system for invasive lung adenocarcinoma, as described in the latest publication of the 2021 edition of the WHO classification of chest tumors, are shown in Table 1^5^. This criterion was proposed by the pathology committee of the International Association for Study of Lung Cancer (IASLC)^6^ and was also adopted by the Chinese Medical Association. As pathological images become increasingly important and abundant, pathologists face growing pressure in diagnosis. The demand for computer-aided diagnostic models in the medical system continues to rise. Therefore, the use of computer-assisted diagnostic models to assist pathologists in diagnosing lung adenocarcinoma grades that have small interclass and large intraclass differences has an important application value.

**Table 1:** Grading scheme for invasive pulmonary adenocarcinomas^6^.

Grade (differentiation)	Patterns
1 (well)	Lepidic predominant with no or less than 20% of high-grade patterns
2 (moderately)	Acinar or papillary predominant with no or less than 20% of high-grade patterns
3 (poorly)	Any tumor with 20% or more of high-grade patterns

The model is based on the predominant histologic plus high-grade patterns. The latter includes solid, micropapillary, and complex glandular patterns.

However, research on lung adenocarcinoma tissue pathological images^7–9^ primarily focuses on intelligent classification of pathological types, while overlooking the study of pathological grading tasks, which to some extent hinders the formulation of personalized treatment plans for physicians. Currently, there is often a problem of large intra-class differences and small inter-class differences between pathological images of lung adenocarcinoma tissues under different grades. If attention mechanisms such as Coordinate Attention (CA)^10^ are directly used for lung adenocarcinoma grading tasks, two issues may arise: CA only extracts global features through average pooling, thus risking excessive compression of feature information, affecting the model's ability to compare local features of lung adenocarcinoma; CA processes too few operations within the same dimension, lacking the process of embedding interactions within the same dimension, thus affecting the model's perception of global information in lung adenocarcinoma tissue pathological images. Therefore, to address the challenge of difficulty in grading lung adenocarcinoma, we propose Dimension Information Embedding Attention Net (DIEANet), which enables the model to focus on the local growth morphology of individual cells while also considering the overall growth pattern of cell clusters. Specifically, our contributions are as follows:

1. We integrate different pooling techniques to automatically select local regions representing key growth patterns of lung adenocarcinoma cells, enhancing the model's attention to local information.
2. We employ an interactive fusion approach to focus on feature information within the same dimension and across dimensions, improving the model's capacity to incorporate dimension information and extract remote dependency relationships. This enables the model to better consider the overall growth patterns of cell clusters.
3. In terms of objective metrics, DIEANet achieved an accuracy of 88.19%, an AUC of 96.61%, an MCC of 81.71%, and a Kappa of 81.16%. Compared to other 7 attention mechanisms, DIEANet achieved state-of-the-art objective metrics, and it aligns better with the visual attention of pathologists under subjective observation.

The following sections are structured as follows: In Section “Related work”, we delve into the evolution of deep learning techniques for lung cancer diagnosis in recent years, and the application and development of Coordinate Attention (CA). Section “Methods” describes the structure of DIEANet in detail. Section “Experiments” shows the lung adenocarcinoma dataset and experimental setup, including comparison experiments and ablation experiments. Section “Summary and outlook” makes some summary and outlook.

## Related work

Currently, research in computer-aided diagnosis of lung cancer mainly focuses on the classification tasks of subtypes such as adenocarcinoma (LUAD), squamous carcinoma (LUSC), and large cell carcinoma. In 2017, Teramoto et al.^11^ correctly classified 71% of cytological pictures of lung cancer into the three lung cancer subtypes LUAD, LUSC, and small cell carcinoma using triple cross-validation to train Deep Convolution Neural Network (DCNN). In 2018, Coudray et al.^12^ and Khosravi et al.^13^ both published a paper on deep learning-based classification for LUAD and LUSC, where the former demonstrated that deep learning can effectively help pathologists detect lung cancer subtypes and even genetic mutations, and the latter investigated the effect of fine-tuning pre-trained CNN models on classification. In 2020, Moitra et al.^14^ used a one-dimensional CNN model to study the staging and grading of non-small cell carcinoma (NSCLC) based on CT images and clinical data, and they achieved good results but did not collect important histopathological images. In addition to improving model performance, in 2022, Civit-Masot et al.^9^ turned their attention to the interpretability aspect, and the proposed system can output the diagnostic results, as well as the image regions focused by the model, which can be used to provide more supporting information to physicians. This inspires us to enhance the model's focusing ability on the most densely populated regions of cancer cells with the highest malignancy level through attention mechanisms.

The aforementioned studies only consider the task of classifying different types of lung cancer, but overlook the significance of different grading of lung adenocarcinoma for personalized treatment and prognosis management. Identifying different grades of lung adenocarcinoma requires models to discern the growth patterns of cells more intricately, focusing on both the local growth morphology of individual cells and the overall growth pattern of cell clusters. We found that Coordinate Attention (CA) proposed by Hou et al.^10^ not only captures inter-channel information but also includes directional and dimensional information, which can enhance the network's vision and performance. This aligns with our approach; hence, we have organized and summarized CA as well as its applications and developments in recent years, as shown in Table 2.

**Table 2:** Coordinate attention (CA) and its applications and developments.

Model name	Authors and date	Summarize
Coordinate attention (CA)	Hou et al. (2021)	Incorporating coordinate information into the channel attention mechanism enhances both the visual acuity and overall performance of the network, all the while maintaining a low computational burden and minimizing parameter count
Sparse label assignment (SLA)^15^	Ming et al. (2021)	Applying CA for object localization in aerial imagery
MSF-MIF^16^	Yang et al. (2022)	Utilizing the CA module to aid CNN in distinguishing objects in hyperspectral images
Attention-augmented grasp detection network (AAGDN)^17^	Zhou et al. (2023)	Combining CA with feature fusion and pyramid modules yielded promising results in object detection experiments
Efficient multi-scale attention (EMA)^18^	Ouyang et al. (2023)	Improvements were made to address channel dimension reduction in CA by grouping features at the channel level and aggregating them with cross-scale interaction methods. Results in classification and object detection tasks validate the effectiveness of this approach

Different from the above studies, we are concerned with the problems of severely compressed information and insufficient calculation of information relevance within the same dimension in CA. These problems somewhat reduce the degree of dimensional information embedding, and cannot be well solved by the problem of large intra-class differences and small inter-class differences that exist in lung adenocarcinoma histopathology images. Therefore, this paper proposes the DIEANet structure for lung adenocarcinoma grading, starting from two aspects: extracting more effective feature information and embedding dimensional information more comprehensively. In the following, we will provide a detailed description of DIEANet.

## Methods

### Overview

**Figure 1:**
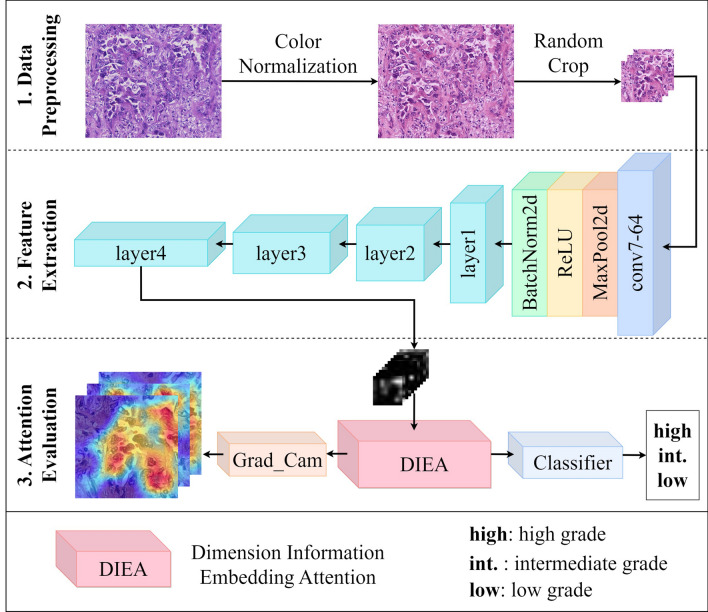
Dimension Information Embedding Attention Net (DIEANet).

The complete structure of DIEANet is illustrated in Fig. 1, which can be divided into three stages. The first stage is the data preprocessing stage, including color normalization and random cropping of histopathological images, which will be detailed in Section “Preprocessing”. The second stage is the feature extraction stage, where ResNet34 is utilized for feature extraction. The specific code uses the ResNet34 code provided by the torchvision library in the Pytorch platform. The third stage is the attention evaluation stage, consisting of Dimension Information Embedding Attention (DIEA), a classifier, and Grad_Cam, which will be elaborated on in Section “Dimension information embedding attention (DIEA)”. This stage validates the performance of DIEANet through both quantitative metrics and visualization.

### Dimension information embedding attention (DIEA)

**Figure 2:**
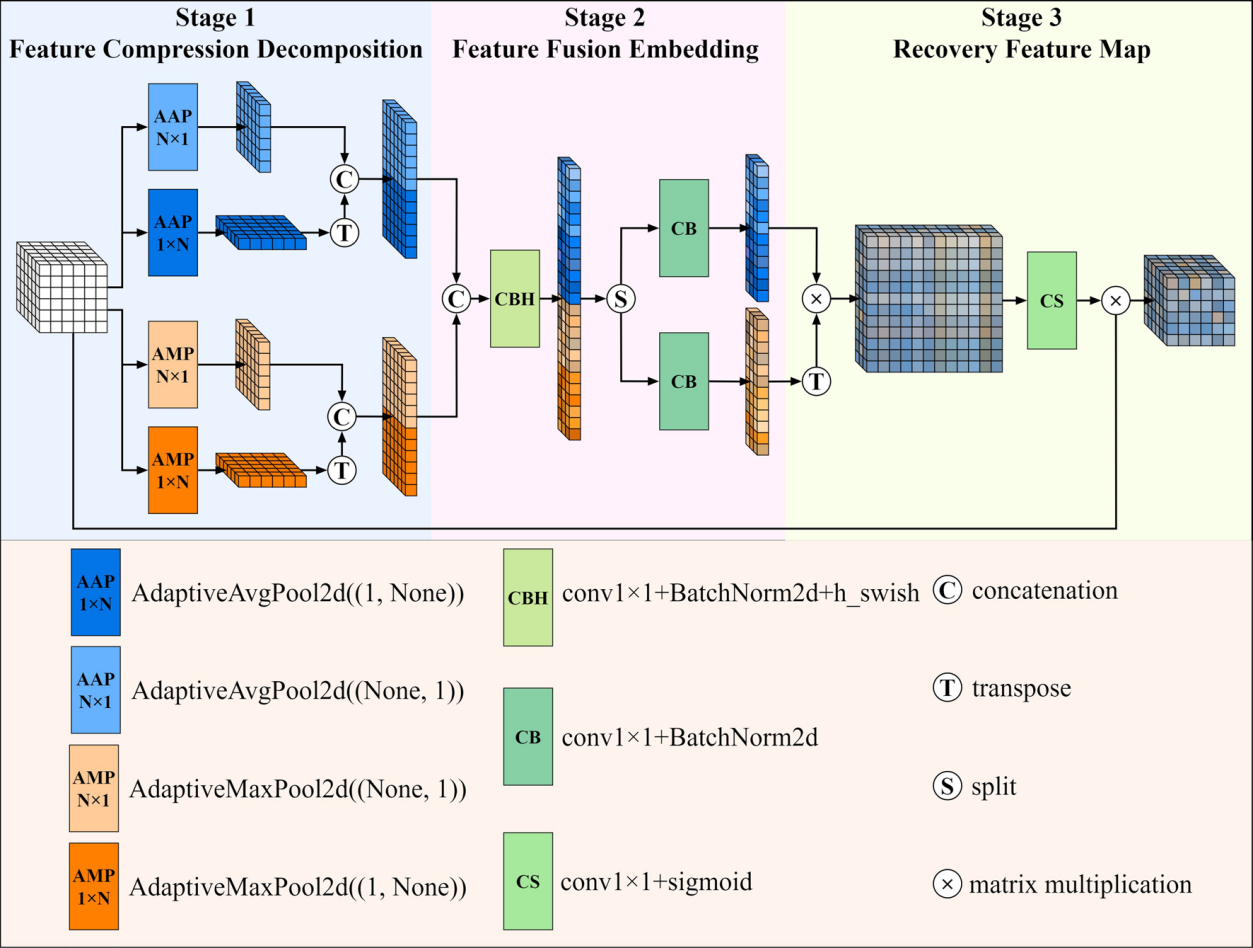
Dimension information embedding attention (DIEA).

To boost the model's efficacy and elevate focus on lesion sites, we propose the DIEA structure. Within DIEA, it specifically includes operations such as feature compression decomposition, feature fusion embedding processing, and restored feature map operations. The detailed structural diagram is shown in Fig. 2.

#### Feature compression decomposition stage

First, the formula for the feature compression decomposition operation is expressed as follows:

1 $$a_{c}^{h} (h) = \frac{1}{W}\sum\limits_{0 \le i < W} {x_{c} } (h,i)$$

2 $$a_{c}^{w} (w) = \frac{1}{H}\sum\limits_{0 \le j < H} {x_{c} } (j,w)$$

3 $$m_{c}^{h} (h) = max_{0 \le i < W} x_{c} (h,i)$$

4 $$m_{c}^{w} (w) = max_{0 \le j < H} x_{c} (j,w)$$

where ${\mathbf{x}}$ denotes the input features, $h$ and $w$ the indexes of feature map height and width, respectively. $a_{c}^{h} (h)$, $a_{c}^{w} (w)$ denote the features obtained by strip average pooling in horizontal and vertical dimension, respectively. $m_{c}^{h} (h)$, $m_{c}^{w} (w)$ denote the features obtained by strip max pooling in horizontal and vertical dimension, respectively. After that, the four 1D feature tensors are combined two by two according to the pooling method to obtain two 1D feature description tensors of length $(H + W)$:

5 $${\mathbf{a}} = [{\mathbf{a}}^{h} ,({\mathbf{a}}^{w} )^{T} ]$$

6 $${\mathbf{m}} = [{\mathbf{m}}^{h} ,({\mathbf{m}}^{w} )^{T} ]$$

where $T$ denotes the transpose operation and [*,*] denotes the splicing operation. In this stage, ${\mathbf{a}}$ containing the remote dependencies of features, ${\mathbf{m}}$ highlighting the features of the key growth morphology of cancer cells, and the combination of the two can provide more effective feature information for the feature embedding operation later in the model. The experimental results in Section “Experimental results and comparison experiments” demonstrate this point.

#### Feature fusion embedding processing

In the second stage, in order to better fuse the features with each other, we stitch ${\mathbf{a}},{\mathbf{m}}$ together to form a one-dimensional feature description tensor of length $(2H + 2W)$, which is separated after nonlinear processing. The process is represented as follows:

7 $${\mathbf{h}} = \delta (BN(F_{1} ([{\mathbf{a}},{\mathbf{m}}])))$$

8 $${\mathbf{h}}^{a} = BN(F_{2} ({\mathbf{h}}_{{\left[ {0:(H + W)} \right]}} ))$$

9 $${\mathbf{h}}^{m} = BN(F_{3} (({\mathbf{h}}_{{\left[ {(H + W):(2H + 2W)} \right]}} )^{T} ))$$

$F_{1} (),F_{2} (),F_{3} ()$ denotes the $1 \times 1$ convolution kernel, $BN()$ denotes Batch-Normalization, and $\delta ()$ denotes the h_swish activation function. H_swish is proposed by MobileNetV3^19^ and is a combination of ReLU and linear functions. The convolution operation of $F_{1} ()$ uses the squeeze operation of the SE block to perform channel dimensionality reduction using the $1 \times 1$ convolution kernel, and the resulting tensor is ${\mathbf{h}} \in {\mathbb{R}}^{C//r \times (2H + 2W) \times 1}$. ${\mathbf{h}}^{a} \in {\mathbb{R}}^{C \times (H + W) \times 1} ,{\mathbf{h}}^{m} \in {\mathbb{R}}^{C \times 1 \times (H + W)}$ denote two one-dimensional feature description tensor of length $(H + W)$.

#### Recovery feature map processing

**Figure 3:**
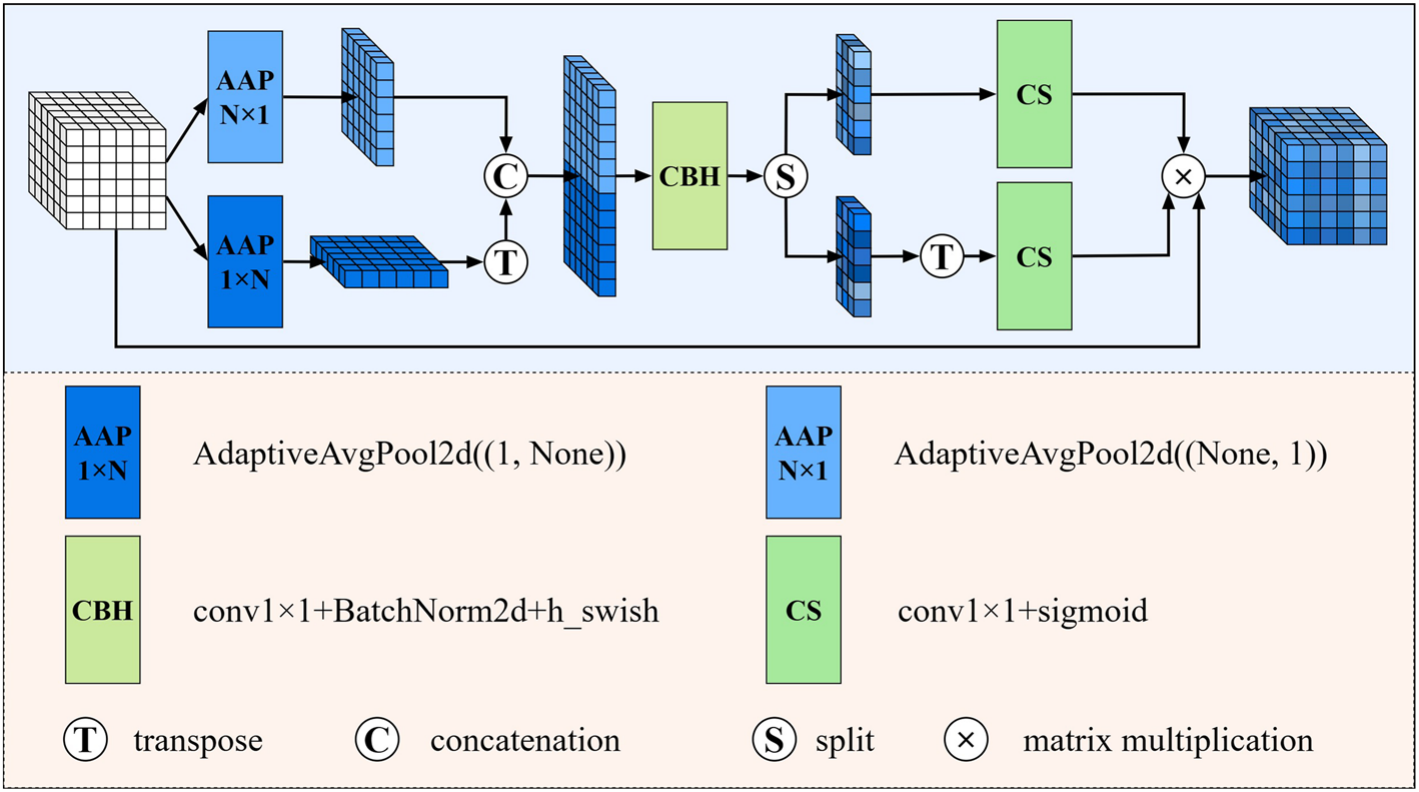
Coordinate attention (CA).

In the third stage, in order to recover the feature map, we multiply ${\mathbf{h}}^{a}$ and ${\mathbf{h}}^{m}$ to obtain the feature map ${\mathbf{h}}^{am} \in {\mathbb{R}}^{C \times (H + W) \times (H + W)}$ containing dimensional embedding information, and this part of the operation is expressed as:

10 $${\mathbf{h}}^{am} = {\mathbf{h}}^{a} \times {\mathbf{h}}^{m}$$

where ${\mathbf{h}}^{a}$ and ${\mathbf{h}}^{m}$ contain the transformed features $\left[ {{\mathbf{a}}^{h} ,({\mathbf{a}}^{w} )^{T} } \right]$ and $\left[ {({\mathbf{m}}^{h} )^{T} ,{\mathbf{m}}^{w} } \right]$, respectively, and after multiplying them, the feature map ${\mathbf{h}}^{am}$ will contain four kinds of information, i.e., $({\mathbf{a}}^{h} \times ({\mathbf{m}}^{h} )^{T} )$, $({\mathbf{a}}^{h} \times {\mathbf{m}}^{w} )$, $(({\mathbf{a}}^{w} )^{T} \times ({\mathbf{m}}^{h} )^{T} )$, and $(({\mathbf{a}}^{w} )^{T} \times {\mathbf{m}}^{w} )$. In contrast, CA generates only one kind of information $({\mathbf{a}}^{h} \times {\mathbf{a}}^{w} )$ when recovering the feature map, and although it achieves the inter-embedding of features in both horizontal and vertical dimensions, it ignores the inter-embedding of features within the same dimension, such as between horizontal and horizontal and between vertical and vertical. To better distinguish between DIEA and CA, the structure of CA is shown in Fig. 3.

Finally, ${\mathbf{h}}^{am}$ is downsampled and embedded in the input feature ${\mathbf{x}}$. The equation is expressed as follows:

11 $${\mathbf{g}} = \sigma (F \downarrow ({\mathbf{h}}^{am} ))$$

12 $${\mathbf{y}} = {\mathbf{x}} \times {\mathbf{g}}$$

where $F \downarrow ()$ denotes a convolutional kernel with kernel size of 3 × 3, stride of 2, and padding of 1. $\sigma$ represents the sigmoid, and ${\mathbf{g}} \in {\mathbb{R}}^{C \times H \times W}$ is the same size as the input feature ${\mathbf{x}}$. ${\mathbf{y}}$ is the final attention-enhanced feature map.

### Ethics approval

This study was approved by the Medical Ethics Committee of the Key Laboratory of Kelamayi Central Hospital on February 10th, 2023.The the ethics approval number is "K202302-29". The informed consent was obtained from the subjects.

## Experiments

### Dataset

We utilized two datasets to assess the model's performance. A private dataset was employed for a thorough examination within the paper's core application domain, while a publicly accessible dataset was utilized to validate the model's effectiveness and its capacity for generalization.

Dataset for lung adenocarcinoma histopathological image grading: This dataset was provided by the Karamay Central Hospital of Xinjiang and approved by the Medical Ethics Committee of the Key Laboratory of Kelamayi Central Hospital, and contains three labels of low-grade, intermediate-grade, and high-grade lung adenocarcinoma. All experiments adhered to pertinent guidelines and regulations. All cases were diagnosed between November 2016 and August 2019, and were confirmed by histopathological examination and informed consent was obtained from the participants. The dataset contains a total of 107 cases, including 30 cases of low grade, 38 cases of intermediate grade, and 39 cases of high grade. The dataset was all stained by hematoxylin–eosin staining (HE staining), and the grading labels of the images were retrieved from the hospital's data system. Since cancer cells coexist in multiple growth patterns in lung adenocarcinoma, for example, there may be a small number of low grade growth patterns in intermediate grade lung adenocarcinoma, we asked two experienced pathologists from the Karamay Central Hospital to draw a finer representative lesion area with the highest and most intensive malignancy for each case as a way to obtain more accurate images of the lesions. The digital slice scanning system PRECICE 500b was then used to scan within the range marked by the physicians. Depending on the size of the lesion area marked by the physicians in the section, we scanned 2–10 random images in jpg format from each case, each with a size of 1665 × 1393 px. A total of 893 images were obtained, including 260 low grade, 316 intermediate grade, and 317 high grade images.

PCam200^20^: This dataset is derived from the Camelyon2016 challenge dataset^21^ by sliding window extraction, used to train models to detect the presence of lymph node metastasis. Tumor patches and normal patches are respectively cropped from annotated regions of tumor slides and tissue regions of normal slides. Subsequently, the cropped patch sizes are adjusted to 512 × 512 pixels using bicubic interpolation. After balancing the number of tumor and normal samples, a total of 56,703 images are randomly split into training, validation, and testing sets, where patches from the same slide are placed in the same set (slide-level splitting). Detailed information about the dataset is shown in Table 3.

**Table 3:** Composition of the PCam200 dataset.

Classes	Training	Validation	Test	Image size
2	28,539	10,490	17,674	512 × 512 px

"Classes" represents the number of image categories included in the dataset. "Training," "Validation," and "Test" denote the number of images in each respective set.

### Preprocessing

**Figure 4:**
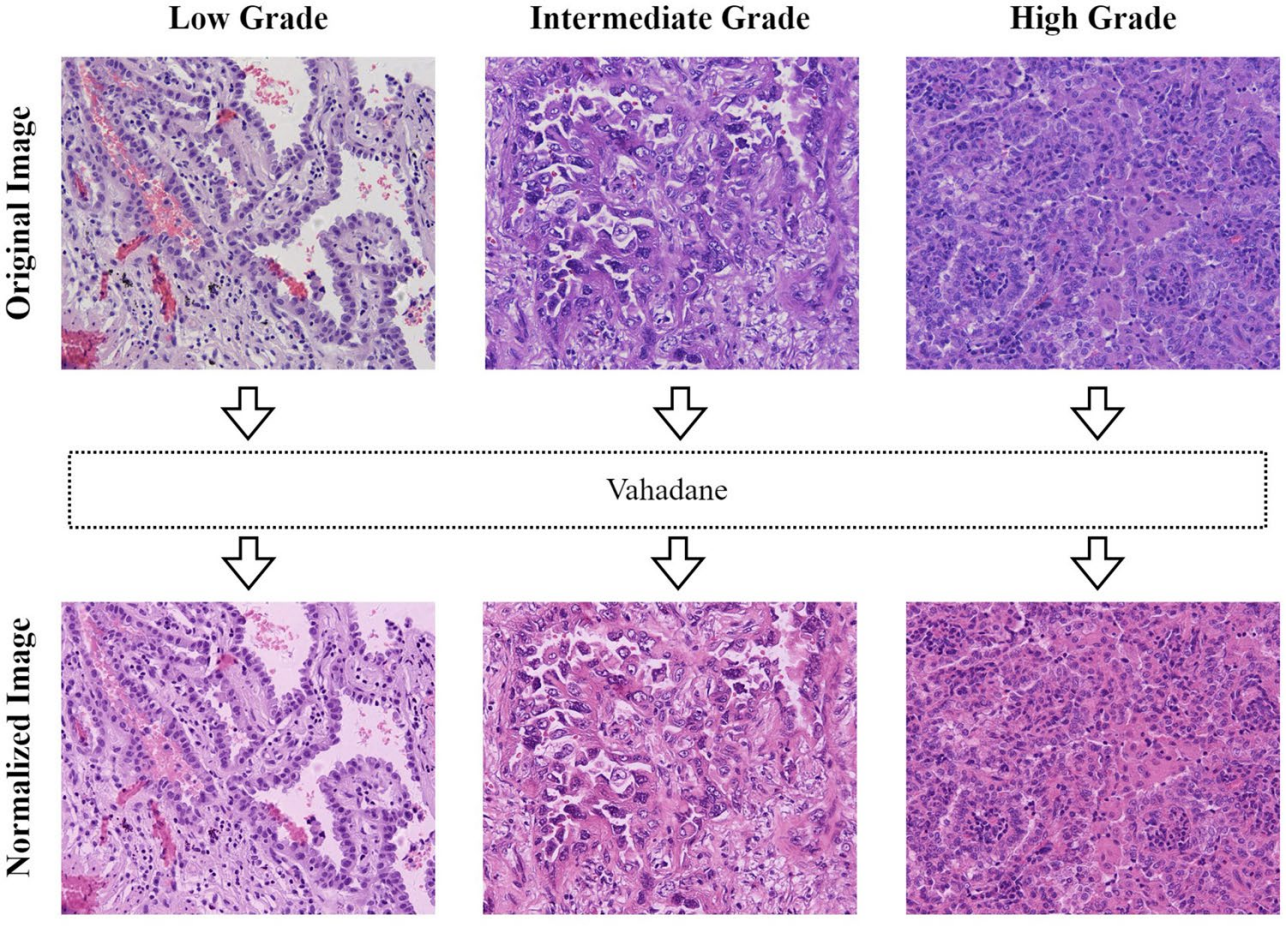
Stain normalization effect. The top row is the original image, the bottom row is the image after Vahadane processing.

We observed excessive color differences between the HE-stained histopathology images and therefore used the currently more popular Vahadane^22^ method for stain normalization of the dataset, the results of which are shown in Fig. 4.

We referenced common processing methods for histopathological image datasets as well as the training approach of cross-validation^23,24^. Initially, at the case level, we utilized five-fold cross-validation to partition the dataset into five folds. Then, we extracted 3 images per normalized image through random cropping, ensuring that all images from the same case are used only for training or validation. Besides, in order to ensure experimental rigor, we removed the images with blank parts accounting for more than 75% of the overall. The final number of images in each category is shown in Table 4.

**Table 4:** Composition of lung adenocarcinoma dataset.

Category	Case load	Original data	Enhanced data
Low grade	30	260	779
Intermediate grade	38	316	945
High grade	39	317	942
Total	107	893	2666
Data size (px)	–	1665 × 1393	256 × 256

Case Load represents number of patients. Original Data represents number of pictures scanned by digital slice scanning system in each category. Enhanced Data represents number of pictures in each category after random cropping.

### Experimental setup details

The experiments were taken with Python version 3.7.13, torch version 1.10.2 + cu102, and GPU V100-PCIE-16 GB. The batch size of the experiment is set to 16, the input image size is set to 256 × 256 px, the optimizer adopts AdamW, the learning rate is 1e-4, while the weight decay is set to 1e−2. The learning rate adjustment strategy is set to use linear learning rate warm-up at the first epoch, and cosine annealing strategy is used for 1–200 epoch to gradually reduce the learning rate to 0 according to cosine function. Cross Entropy Loss, which is commonly used in classification model, is used for training to converge the model.

For the lung adenocarcinoma grading dataset, we employed stratified five-fold cross-validation during model training, with each fold trained for 200 epochs. At the case level, we randomly divided patients into five folds, and when reading images, we determined their belonging to each fold based on their pathological identification number. This approach ensures both balanced data in training and validation sets and independence between them, thereby ensuring the authenticity of the experimental results.

For the PCam200 dataset, we train 200 epochs according to the training, validation, and test sets that have been divided by the authors.

### Experimental results and comparison experiments

We selected three backbone frequently used in cancer pathology image classification tasks to validate the performance of DIEA, namely ResNet34^25–27^, MobileNetV2^28,29^, and EfficientNet^30,31^. The pre-trained weights obtained from ImageNet training provided in torchvision.models were used in all training.

To measure the performance of the model in multiple aspects, a total of nine metrics were used to evaluate the model performance in two aspects. First, in terms of computational complexity, Number of Parameters (Params) and Giga Floating Point Operations per Second (GFLOPs) are used. These two metrics are used to measure the computational resource requirements and computational efficiency of the model. In this paper, both Params and GFLOPs are computed with the input image size of 256 × 256 px. Second, for the performance metrics, we used Accuracy (ACC), F1-Score (F1), Precision (PRE), Recall, Area Under Curve (AUC), Matthew's Correlation Coefficient (MCC), Cohen's Kappa score (Kappa) which are seven metrics^32,33^. These metrics are used to assess the performance and prediction quality of the model. ACC measures the overall prediction accuracy of the model, F1 combines PRE and Recall, while PRE and Recall measure the positive case prediction accuracy and positive case prediction ability of the model, respectively. Furthermore, given that the model's task involves three-class classification, we considered two averaging methods to comprehensively showcase the model's performance. "_m" in the table indicates macro-average, where all classes are equally weighted without considering sample distribution. "_w" denotes weighted-average, providing a more accurate reflection of overall classification performance. AUC measures the performance of the classification model, and MCC and Kappa score are used to assess the prediction quality and consistency of the classification model. The experimental results display the averages and standard deviations of five-fold cross-validation.

With ResNet34 as the backbone, the comparison results of DIEANet with SimAM^34^, BAM^35^, Criss-Cross Attention (CCA) in CCNet^36^, CBAM^37^, CA, EMA^38^, ACmix^18^ which are seven types of attention, are shown in Table 5.

**Table 5:** Comparison experiments with different attentions when ResNet34 is the backbone, where the bolded numbers are the highest scores in a column and the underline indicates the second highest in a column.

	Backbone (ResNet34)	Backbone + SimAM	Backbone + BAM	Backbone + CCA	Backbone + CBAM	Backbone + CA	Backbone + EMA	Backbone + ACmix	DIEANet (ours)
Params	21.32M	21.32M	21.39M	21.65M	21.35M	21.34M	21.32M	22.15M	21.4 M
FLOPs	4.8042G	4.8042G	4.8065G	4.8252G	4.8043G	4.8046G	4.8092G	4.8582G	4.8093G
ACC	87.03 ± 5.04	86.94 ± 4.76	87.23 ± 4.35	86.89 ± 4.84	87.44 ± 3.78	87.90 ± 4.82	87.36 ± 4.79	87.37 ± 4.56	**88.19** ± 4.26
AUC	95.90 ± 2.92	95.92 ± 2.24	95.87 ± 2.60	95.82 ± 2.48	96.33 ± 1.68	96.09 ± 2.15	96.00 ± 2.54	95.87 ± 2.21	**96.61** ± 2.11
MCC	80.26 ± 7.40	79.90 ± 6.87	80.37 ± 6.44	79.81 ± 7.29	81.13 ± 5.59	81.19 ± 6.99	80.76 ± 6.84	80.35 ± 6.96	**81.71** ± 6.24
Kappa	79.58 ± 7.69	79.25 ± 7.35	79.75 ± 6.82	79.23 ± 7.53	80.35 ± 5.79	80.77 ± 7.31	80.00 ± 7.36	79.79 ± 7.14	**81.16** ± 6.66
F1_m	85.68 ± 5.49	84.99 ± 5.72	85.74 ± 5.30	85.79 ± 5.27	85.91 ± 3.99	**86.73** ± 5.16	85.58 ± 5.44	86.08 ± 4.95	86.40 ± 4.43
PRE_m	86.89 ± 5.12	86.44 ± 5.02	87.04 ± 4.63	87.40 ± 4.68	87.36 ± 3.60	**87.95** ± 4.67	86.76 ± 4.74	87.54 ± 4.69	87.55 ± 4.25
Recall_m	87.32 ± 5.45	86.47 ± 5.12	87.24 ± 4.89	87.05 ± 4.88	87.49 ± 3.69	87.90 ± 4.72	87.60 ± 5.06	87.39 ± 4.36	**88.08** ± 4.14
F1_w	86.86 ± 5.16	86.66 ± 5.02	87.08 ± 4.47	86.71 ± 4.98	87.24 ± 3.88	87.87 ± 4.83	87.17 ± 4.99	87.26 ± 4.53	**88.13** ± 4.26
PRE_w	89.18 ± 4.74	88.79 ± 4.11	89.28 ± 3.74	88.87 ± 4.25	89.78 ± 3.34	89.78 ± 3.96	89.58 ± 4.19	89.42 ± 3.83	**90.29** ± 3.65
Recall_w	87.03 ± 5.04	86.94 ± 4.76	87.23 ± 4.35	86.89 ± 4.84	87.44 ± 3.78	87.90 ± 4.82	87.36 ± 4.79	87.37 ± 4.56	**88.19** ± 4.26

All performance metrics in %.

According to Table 5, our model achieved the highest values in 8 out of 10 objective metrics, with the remaining two ranking second, and the standard deviations generally being low. Particularly noteworthy is the five-fold average accuracy for lung adenocarcinoma grading, which reached 88.19% ± 0.0426, demonstrating outstanding performance along with good generalization and robustness.

**Figure 5:**
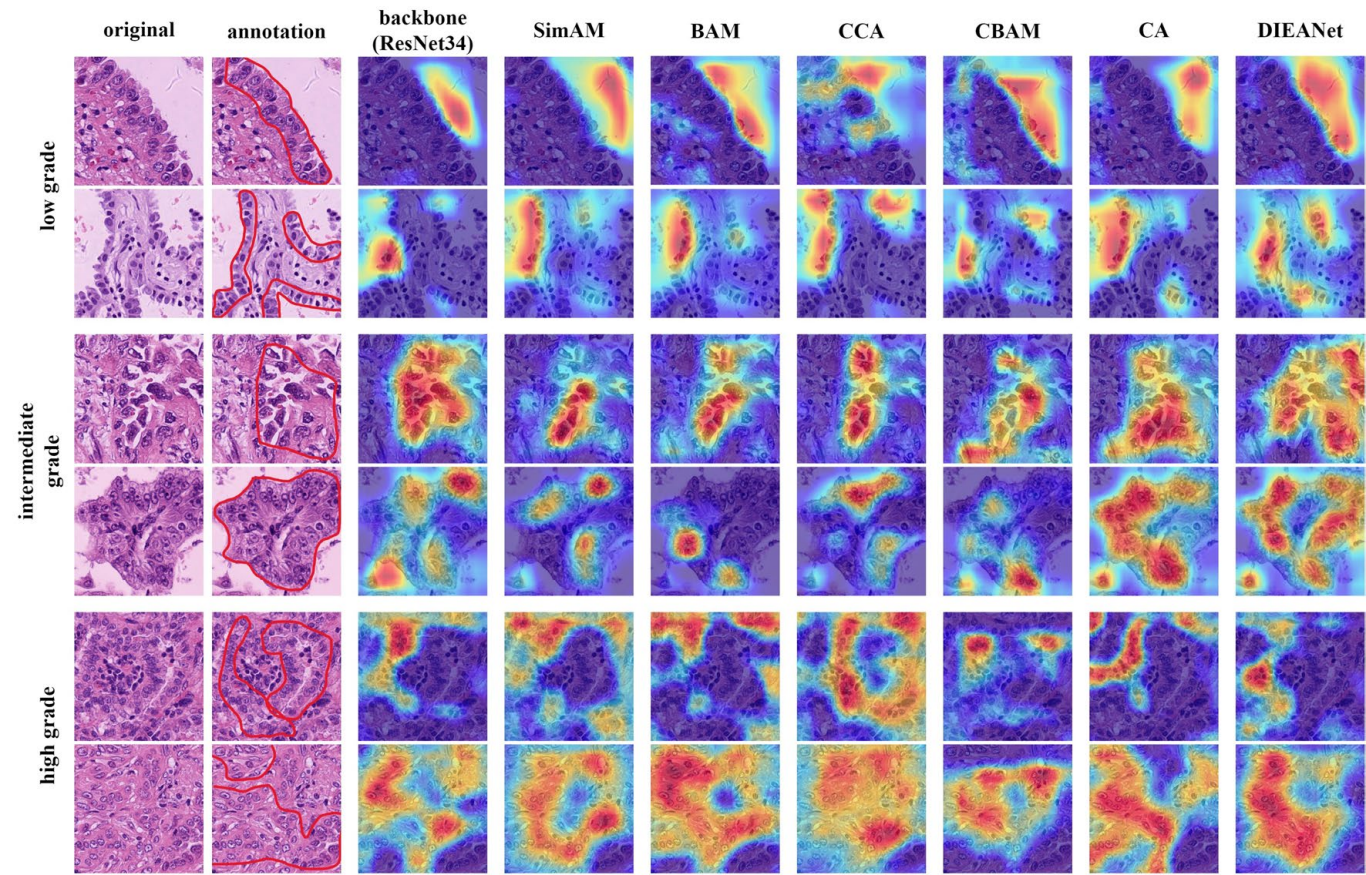
Gradient-weighted class activation map for each model when ResNet34 is the backbone.

We show the gradient-weighted class activation map generated by different attentions in Fig. 5 to analyze the spatial localization ability of DIEANet.

As shown in Fig. 5, DIEANet focuses on a more comprehensive and accurate region, which obviously can better identify global information while paying attention to location information compared to other attention, and can assist pathologist to better locate the region of interest.

We then compared with other attentions again using MobileNetV2 and EfficientNet as backbone, respectively.

According to Table 6, when using MobileNetV2 as the backbone, DIEANet achieved the highest values in all 10 performance metrics, with low standard deviations across the board. The five-fold average accuracy for lung adenocarcinoma grading reached 87.22% ± 0.0444, demonstrating outstanding performance. Similarly, as indicated in Table 7, when employing EfficientNet as the backbone, DIEANet attained the highest values in 9 out of 10 performance metrics, with generally low standard deviations. The five-fold average accuracy for lung adenocarcinoma grading reached 87.70% ± 0.0446, also showcasing remarkable performance.

**Table 6:** Comparison experiments with different attentions when MobileNetV2 is the backbone, where the bolded numbers are the highest scores in a column and the underline indicates the second highest in a column.

	Backbone (MobileNetV2)	Backbone + SimAM	Backbone + BAM	Backbone + CCA	Backbone + CBAM	Backbone + CA	Backbone + EMA	Backbone + ACmix	DIEANet (ours)
Params	2.23M	2.23M	2.65M	4.28M	2.43M	2.38M	2.24M	7.25M	2.74M
FLOPs	0.4261G	0.4261G	0.4403G	0.5571G	0.4265G	0.4279G	0.4567G	0.7496G	0.4574G
ACC	85.71 ± 4.90	86.51 ± 4.65	86.03 ± 5.47	85.77 ± 4.99	86.79 ± 4.60	86.49 ± 4.72	86.91 ± 4.41	85.98 ± 5.16	**87.22** ± 4.44
AUC	94.94 ± 2.61	95.72 ± 2.21	95.24 ± 2.78	95.05 ± 3.03	95.45 ± 2.37	95.60 ± 3.04	95.59 ± 2.31	95.50 ± 2.14	**96.01** ± 2.33
MCC	77.26 ± 6.18	79.43 ± 6.46	78.68 ± 7.90	78.48 ± 7.08	79.29 ± 6.88	79.38 ± 6.59	79.78 ± 6.29	78.97 ± 7.23	**79.89** ± 7.48
Kappa	77.13 ± 7.29	78.67 ± 7.06	77.98 ± 8.33	77.61 ± 7.59	78.64 ± 7.25	78.52 ± 7.22	79.29 ± 6.74	78.09 ± 7.75	**79.74** ± 7.10
F1_m	83.95 ± 5.29	84.69 ± 5.17	84.57 ± 6.01	83.93 ± 5.44	84.86 ± 5.69	84.88 ± 5.36	84.77 ± 4.88	84.73 ± 5.55	**85.99** ± 4.79
PRE_m	85.25 ± 5.09	86.06 ± 4.58	86.03 ± 5.61	85.25 ± 4.88	86.09 ± 5.32	86.69 ± 4.06	85.51 ± 4.32	86.34 ± 5.20	**87.14** ± 4.48
Recall_m	85.45 ± 5.16	86.44 ± 4.39	86.02 ± 5.35	85.41 ± 4.93	86.32 ± 5.32	86.47 ± 4.97	86.56 ± 4.53	86.52 ± 4.58	**87.56** ± 4.52
F1_w	85.63 ± 5.03	86.29 ± 4.77	85.70 ± 5.71	85.59 ± 5.30	86.60 ± 4.64	86.18 ± 5.13	86.67 ± 4.68	85.63 ± 5.40	**87.13** ± 4.42
PRE_w	87.82 ± 4.67	88.71 ± 3.60	87.88 ± 4.99	88.05 ± 4.46	88.56 ± 3.97	88.83 ± 3.96	88.44 ± 4.11	88.19 ± 4.54	**89.32** ± 3.87
Recall_w	85.71 ± 4.90	86.51 ± 4.65	86.03 ± 5.47	85.77 ± 4.99	86.79 ± 4.60	86.49 ± 4.72	86.91 ± 4.41	85.98 ± 5.16	**87.22** ± 4.44

All performance metrics in %.

**Table 7:** Comparison experiments with different attentions when EfficientNet is the backbone, where the bolded numbers are the highest scores in a column and the underline indicates the second highest in a column.

	Backbone (EfficientNet)	Backbone + SimAM	Backbone + BAM	Backbone + CCA	Backbone + CBAM	Backbone + CA	Backbone + EMA	Backbone + ACmix	DIEANet (ours)
Params	4.01M	4.01M	4.44M	6.06M	4.22M	4.17M	4.03M	9.04M	4.53M
FLOPs	0.5404G	0.5404G	0.5546G	0.6714G	0.5408G	0.5422G	0.5710G	0.8639G	0.5717G
ACC	86.94 ± 4.91	86.53 ± 4.93	86.92 ± 4.64	86.25 ± 4.80	87.51 ± 4.44	87.21 ± 4.54	87.32 ± 4.47	86.97 ± 4.21	**87.70** ± 4.46
AUC	95.90 ± 1.91	95.87 ± 2.42	95.73 ± 2.18	95.37 ± 2.53	95.83 ± 1.86	**96.29** ± 2.03	96.06 ± 2.44	95.93 ± 1.72	95.82 ± 2.73
MCC	79.97 ± 7.46	79.53 ± 7.36	79.67 ± 7.11	78.90 ± 7.07	80.52 ± 6.89	79.68 ± 6.83	80.38 ± 6.38	80.13 ± 6.44	**81.28** ± 6.72
Kappa	79.36 ± 7.72	78.75 ± 7.55	79.04 ± 7.42	78.24 ± 7.20	80.08 ± 7.12	79.63 ± 6.91	79.67 ± 6.94	79.40 ± 6.62	**80.66** ± 6.88
F1_m	85.78 ± 5.34	85.14 ± 5.27	85.42 ± 4.99	84.69 ± 4.72	86.34 ± 4.96	85.67 ± 4.47	85.95 ± 4.93	85.71 ± 4.51	**86.60** ± 4.77
PRE_m	87.04 ± 4.63	86.55 ± 4.70	86.79 ± 4.40	85.74 ± 4.37	87.42 ± 4.66	86.78 ± 4.46	87.54 ± 4.02	86.91 ± 4.20	**88.08** ± 4.20
Recall_m	87.36 ± 4.75	86.85 ± 4.79	86.94 ± 4.54	86.31 ± 4.55	87.70 ± 4.54	86.99 ± 4.02	87.32 ± 4.52	87.37 ± 3.94	**88.00** ± 4.61
F1_w	86.78 ± 5.00	86.45 ± 5.14	86.84 ± 4.66	86.10 ± 4.85	87.44 ± 4.49	87.02 ± 4.67	87.02 ± 4.72	86.99 ± 4.22	**87.69** ± 4.43
PRE_w	88.98 ± 4.00	89.05 ± 4.29	89.13 ± 3.76	88.29 ± 4.12	89.38 ± 3.93	88.90 ± 4.32	89.30 ± 3.65	89.49 ± 3.34	**90.04** ± 3.73
Recall_w	86.94 ± 4.91	86.53 ± 4.93	86.92 ± 4.64	86.25 ± 4.80	87.51 ± 4.44	87.21 ± 4.54	87.32 ± 4.47	86.97 ± 4.21	**87.70** ± 4.46

All performance metrics in %.

In PCam200, we compared the performance of DIEANet with seven other attention mechanisms using ResNet34 as the backbone. The results on the test set are presented in Table 8.

**Table 8:** Performance of different attention mechanisms on PCam200 with ResNet34 as backbone.

	Backbone (ResNet34)	Backbone + SimAM	Backbone + BAM	Backbone + CCA	Backbone + CBAM	Backbone + CA	Backbone + EMA	Backbone + ACmix	DIEANet (ours)
ACC	93.25	93.19	92.03	92.89	92.87	92.46	93.01	93.03	**93.57**
AUC	93.27	93.24	92.04	92.81	92.88	92.43	92.98	93.03	**93.54**
MCC	86.79	86.50	84.54	86.04	86.11	85.18	86.24	86.48	**87.27**
Kappa	85.75	85.61	83.22	84.91	85.05	84.05	85.27	85.34	**86.46**
F1	92.48	92.56	90.93	91.84	91.95	91.50	92.18	92.13	**92.92**
PRE	97.79	96.16	97.74	97.82	97.28	96.82	96.84	**97.83**	96.02
Recall	88.59	90.15	86.02	87.48	88.14	87.75	88.87	88.04	**90.91**

The numbers in bold represent the highest score in each row, while those underlined indicate the second-highest. All performance metrics are in percentage (%).

Based on Table 8, it is evident that our model achieved the highest values in six out of seven metrics, with accuracy reaching 93.57% and recall reaching 90.91%. The significantly higher recall value compared to other models is crucial for reducing false negatives in medical diagnosis. These results further confirm the suitability of DIEANet for processing histopathological images.

### Ablation experiments

We conducted ablation experiments using alone strip average pooling or only strip max pooling to extract features to validate the efficiency of combining strip average pooling with strip max pooling to extract features. The experimental results revealed that fusing the two pooling can facilitate the model to extract more effective feature information, which is advantageous to enhance the network's performance. The experimental results are presented in Tables 9 and 10.

**Table 9:** Ablation experiments, where the bolded numbers are the highest scores in a column.

Settings	Params	FLOPs	ACC (%)	AUC (%)	MCC (%)	Kappa (%)
AP^a^ + AP	21.40M	4.8094G	87.34	96.06	80.67	80.12
MP^b^ + MP	21.40M	4.8092G	87.28	95.94	79.99	79.84
AP + MP	21.40M	4.8093G	**88.19**	**96.61**	**81.71**	**81.16**

^a^Average Pooling. ^b^Max Pooling.

**Table 10:** Ablation experiments, where the bolded numbers are the highest scores in a column.

Settings	F1_m	PRE_m	Recall_m	F1_w	PRE_w	Recall_w
AP^1^ + AP	86.23	87.58	87.29	87.34	89.50	87.34
MP^2^ + MP	85.98	87.46	87.60	87.11	89.54	87.28
AP + MP	**86.4**	**87.55**	**88.08**	**88.13**	**90.29**	**88.19**

The _m ending indicates that the averaging approach is macro-average, and the _w ending indicates that the weighted-average approach is used (%).

^a^Average Pooling. ^b^Max Pooling.

## Summary and outlook

In this study, we proposed the Dimension Information Embedding Attention Net (DIEANet) for the task of lung adenocarcinoma histopathological image grading, building upon the improvement of Coordinate Attention (CA). Specifically, DIEANet combines different pooling techniques to automatically select local regions of critical growth patterns such as lung adenocarcinoma cells, enhancing the model's focus on local information. Furthermore, it adopts interactive fusion methods to concentrate on feature information within the same dimension and across dimensions, improving the model's capacity to embed dimensional information and thus improve its perception of global features. Ultimately, extensive experiments demonstrate that, objectively, DIEANet achieves state-of-the-art performance in the grading task of lung adenocarcinoma. Subjectively, it also aligns better with the visual attention of pathology experts. In the future, we will collect pathological images of lung adenocarcinoma tissues from different research institutions to expand our dataset. In addition, considering the significant cost of annotating medical images, we plan to introduce weakly supervised techniques. By learning features from publicly available lung adenocarcinoma pathological images without graded labels, we aim to further enlarge the dataset and enhance model performance.

## Data Availability

The datasets generated and analyzed during the current study are not publicly available due to data privacy laws, but are available from the corresponding author on reasonable request.
